# Signals of Paleotemperature in the Age Structure and Seasonal Distribution of Pollination Syndromes Across a Temperate Region

**DOI:** 10.1002/ece3.74150

**Published:** 2026-08-11

**Authors:** Thomas Leclère, Igor Bartish, Pille Gerhold, Andreas Prinzing

**Affiliations:** ^1^ Institute of Agricultural and Environmental Sciences Estonian University of Life Sciences Tartu Estonia; ^2^ Department of Population Ecology Institute of Botany, Academy of Sciences Prague Czech Republic; ^3^ Research Unit “Ecosystem, Biodiversity, Evolution” Université de Rennes/Centre National de la Recherche Scientifique; Campus de Beaulieu Rennes France

**Keywords:** angiosperm diversification, community assembly, paleoclimate, pollination syndrome, regional flora

## Abstract

Across the planet, angiosperm diversification has been shaped by palaeoclimates and biotic interactions, particularly with pollinators, leaving signatures in the angiosperm age structure. Regional floras represent biased samples of this global diversification shaped by dispersal, extinction and environmental filtering, yet it remains unclear whether floristic age structure reflects pollination strategies or their interaction with palaeoclimates. We constructed a 93% resolved phylogeny of all native 1178 angiosperms in the Netherlands and characterized age structure of eight pollination syndromes by standardized epoch‐specific lineage diversities (stELDs) quantifying the proportional increase in lineages per 10‐Myr interval. Overall, stELDs peaked under the coldest intervals and were lowest for intermediate‐temperature intervals (geological age alone had no effect). This age structure was strongest in wind‐pollinated species. Resolving insect pollination into six syndromes revealed strong heterogeneity: lepidoptera‐pollination was most strongly associated with warmest palaeotemperatures, bumblebee and wasp‐pollination under intermediate temperatures, and fly, lepidoptera and beetle‐pollination under coldest temperatures. Paleotemperature signatures correlated positively with contemporary seasonal flowering temperatures, indicating conserved thermal niches across ecological and macroevolutionary timescales. We suggest that temperate floras reflect both recent cold‐house diversification of wind‐ and certain insect‐pollinated lineages and earlier warm‐house radiations of lineages pollinated by older insect clades. Phylogenetic age structures permit detecting such patterns.

## Graphical abstract

Species with wind & insect pollination syndromes show distinct age structure patterns. Splitting insect pollination into different categories show constrasting age structure. Modern flowering seasons track paleotemperature signatures.

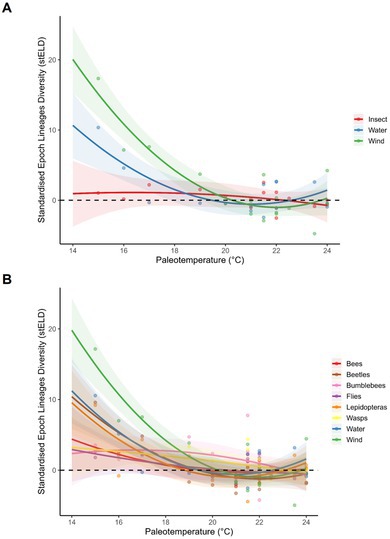

## 1 Introduction

**FIGURE 1:**
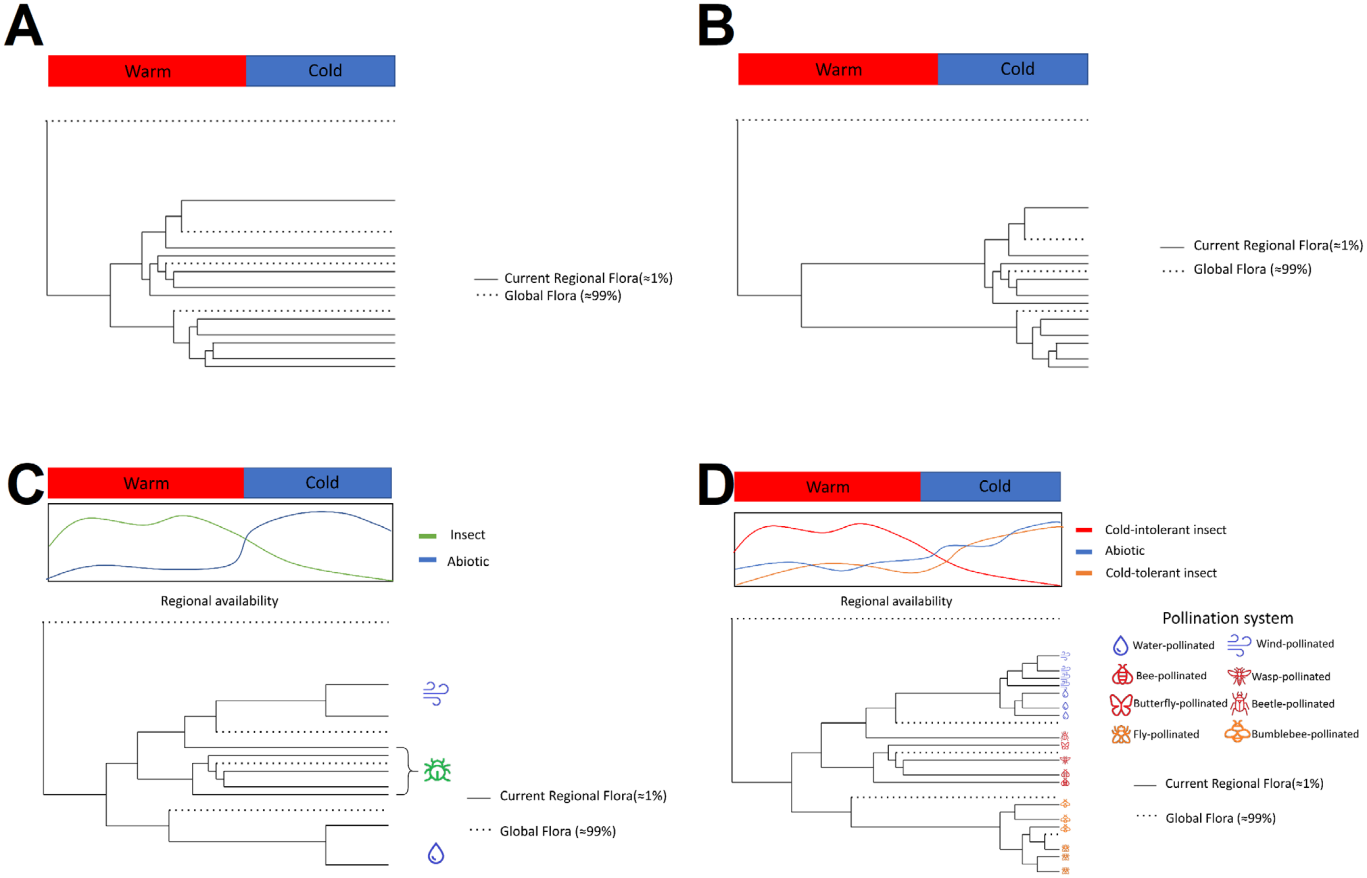
Expected regional phylogenetic age structures under the different hypotheses. (A) Hypothesis (i): Warmer epochs contribute more lineages to the age structure of a regional flora. (B) Hypothesis (ii): Colder epochs contribute more lineages to the age structure of a regional flora. (C) Hypotheses (iii) & (iv): Trees of insect‐pollinated lineages contribute most lineages to overall age structure, while cold epochs contribute most lineages to the age structure of wind‐pollinated species. (D) Hypothesis (v): Cold epochs contribute most lineages to the age structure of wind‐, water‐, fly‐ and bumblebee‐pollinated species, whereas warm epochs contribute most lineages to the age structure of bee‐, wasp‐, lepidoptera‐ and beetle‐pollinated species. Note that the region hosts only less than 1% of the global richness of angiosperms, preventing ancestral‐state reconstruction, but still permitting quantification of the proportional increase in numbers of lineages dating back to a given geological epoch.

Being present since the Mesozoic (Li et al. 2019), angiosperms represent the most evolutionarily and ecologically diverse plant group (Crepet and Niklas 2009; Willis and McElwain 2014). Understanding the historical processes shaping this diversity remains a central question in evolutionary biology. Angiosperm diversification has been driven by both abiotic (e.g., paleoclimate) and biotic interactions that leave distinct traces in the modern age structure of angiosperms. Angiosperm diversification was particularly favored during the warmest geological intervals, when elevated global temperature and high atmospheric CO_2_ concentrations accelerated growth rates, expanded the availability of suitable habitats, and facilitated ecological opportunities for lineage radiation (see Figure 1A for an illustration of the expected age structure) (Zachos et al. 2008; Sauquet and Magallón 2018; Condamine et al. 2013). In addition to these abiotic drivers, biotic interactions have played a central role in shaping angiosperm diversity. Across all epochs, insect‐pollinated lineages are expected to dominate, as coevolution with pollinators has promoted the evolution of diverse floral traits, increased reproductive efficiency, and enabled the occupation of a wide range of ecological niches, enhancing species richness at a global scale (Figure 1C) (Van Der Niet and Johnson 2012; Armbruster 2014; Stephens et al. 2023). Although insect pollination is ancestral and dominated much of angiosperm evolutionary history (Stephens et al. 2023), many insect‐pollinated lineages are comparatively young and often form the most rapidly radiating clades (Van Der Niet and Johnson 2012). These patterns result in a characteristic global age structure of angiosperm lineages, in which older clades persist in climatically stable regions, while bursts of diversification correspond to warming intervals and the expansion of open habitats (Ramírez‐Barahona et al. 2020; Magallón and Castillo 2009). However, global diversification does not translate directly into regional assemblages.

The angiosperms of any given region are a highly biased sample from this global diversification, shaped by dispersal history, local extinction, and environmental filtering. For instance, any given region in the temperate zone is characterized by cool seasonal climates, short growing seasons, and pronounced thermal seasonality (MacColl 2005). Under such conditions, both thermal filtering on plants and climatic constraints on pollinator activity may influence which lineages can persist or diversify regionally. Angiosperm diversification may have been filtered toward lineages originating during cooler geological intervals, reflecting evolutionary constraints on the colonization of cold environments (Figure 1B; Zanne et al. 2014). This pattern is consistent with evidence from northern European floras, where cold‐tolerance filtering has strongly influenced the composition of extant floras over time (Svenning 2003; Svenning et al. 2015). Moreover, in cold climates where insect activity is often limited, wind pollination may provide a more reliable reproductive strategy. Consequently, wind‐pollinated lineages are expected to be proportionally more common across epochs in such environments, given the relative constancy and predictability of wind as a pollen vector under low‐temperature conditions (Friedman and Barrett 2008). On top of these abiotic patterns, different clades of insect pollinators have experienced distinct evolutionary histories, and several old pollinator lineages, such as Lepidoptera, underwent relatively recent diversification during the late Cenozoic cooling and the expansion of temperate and seasonal biomes (Condamine and Hines 2015; Condamine et al. 2020; Rainford and Mayhew 2015; Strömberg 2011). Together, these patterns suggest that for a cool, temperate region, an age structure in which wind‐pollinated lineages and lineages pollinated by recently diversified pollinator lineages tend to dominate across the phylogeny, but this scenario remains largely untested.

Finally, we expect that the age structure depends on the interaction between paleoclimate and pollination strategy: cooler geological epochs should be overrepresented in the age structure of wind pollinated species, because wind pollination remains reliable when insect activity collapses at low temperatures (Figure 1C; Culley et al. 2002; Rafferty and Ives 2011) and is particularly efficient in the open, low‐vegetation landscapes that characterize many cool periods (Friedman and Barrett 2008; Wanntorp and Wanntorp 2003). Consistent with this, geological cooling and the recurrent expansion of open biomes have repeatedly favored the diversification and persistence of wind‐pollinated clades through deep time (Denk et al. 2017; Wang et al. 2025). We may expect the same pattern for the age structure of the second non‐biotic pollination, that is, water pollination. By contrast, age structures of species pollinated by cold‐intolerant insects (e.g., bees (Vilchez‐Russell and Rafferty 2024; Pokhrel 2016), butterflies (Bladon et al. 2020), wasps (Sutton et al. 2018), beetles (Bernhardt 2000)), are expected to be biased toward warm intervals, when insect activity, visitation rates and pollination efficiency peak (Rafferty and Ives 2011). In turn, age structures of species pollinated by cold‐tolerant insects, for example, flies (Lefebvre et al. 2018), bumblebees (Karbassioon et al. 2023) should be centered on colder paleotemperatures, reflecting their ability to forage under cooler conditions (Lefebvre et al. 2018; Karbassioon et al. 2023). Together, these contrasting thermal niches create clear expectations about how different pollination systems should leave distinct temporal signatures in regional age structures (Figure 1D). In a given temperate‐zone region, we would therefore predict epochs of colder paleotemperatures to contribute more lineages to ages structures of wind‐, water‐, fly‐, and bumblebee‐pollinated taxa, whereas lineages pollinated by cold‐intolerant insects should reflect a stronger imprint of warm geological intervals. Although these expectations are consistent with global patterns of pollination ecology and paleoclimate, their manifestation has to our knowledge been empirically tested neither regionally nor globally yet.

Our hypotheses argue that paleotemperatures have influenced the origin of pollination syndromes, and the present phylogenetic age structure of pollination syndromes within a region reflects these origins (rather than later convergence of lineages from cool or warm epochs to different syndromes). At the level of phylogenetic lineages, it is established that the paleotemperatures of origin may today to some extent be reflected in present distributional ranges (Crisp et al. 2009). Grasses, for instance, mostly originated during cool and dry epochs (Strömberg 2011) and today dominate in cool and dry regions. Pollination syndromes are not limited to individual lineages (Fenster et al. 2004; Ashworth et al. 2015), but the many origins of a given syndrome across lineages may nevertheless have happened under specific paleotemperatures, which may still today be reflected in the age to which different pollination syndromes date back, as we hypothesize above. Finally, if paleotemperatures have influenced the origin of pollination syndromes, this may still be reflected in the within‐region distribution of syndromes across temperature gradients in space and time, such as the distribution of flowering activity across seasons. For instance, a syndrome with species dating back to the coldest epochs might today account for most species flowering during the coldest seasons. We hence predict that the relationship between paleotemperatures and the phylogenetic‐age structure of pollination syndromes correlates with the relationship between seasonal temperatures today and the flowering activities of the same pollination syndromes.

To evaluate these predictions, we tested how paleotemperature and pollination strategy together shaped the age structure of angiosperms in one of the best‐characterized regional floras worldwide: the flora of the Netherlands, for which comprehensive information on species‐level phylogeny has recently been produced, and pollination syndromes are available, differentiating even within insects among pollinator groups (contrary to global systems, Stephens et al. 2023). To identify age structure, we use standardized epoch‐specific lineage diversity (stELD, Bartish et al. 2016; Bartish et al. 2020). This metric quantifies for the phylogeny of a given subset of species the proportional increase in the number of lineages dating back to a given past epoch (compared to a null expectation across all subsets), thereby capturing how past diversification events have shaped the age structure of modern floras (Bartish et al. 2016). The metric is applicable where character state reconstruction is not: for entire regional floras, that is, phylogenetically vast but missing almost all species for almost all lineages present. Using stELD, we quantified the numbers of lineages dating back to successive geological epochs for each of eight pollination systems, including insect‐, wind‐ and water‐pollination. Based on the hypotheses developed above, we predicted either (i) a higher overall stELD during warm intervals or (ii) a higher overall stELD during cold intervals; (iii) higher overall stELD in wind‐pollinated species; (iv) higher overall stELD in insect‐pollinated lineages than in wind‐ or water‐pollinated lineages; (v) higher stELD of wind‐pollinated lineages at low paleotemperatures; and (vi) contrasting thermal signatures among insect pollination systems, with flies and bumblebees showing higher stELD at cold or intermediate paleotemperatures, whereas bees, lepidoptera, wasps and beetles should show higher stELD at warm paleotemperatures. We finally described for each pollination syndrome the quadratic relationship between (a) its stELDs across geological epochs and the corresponding paleotemperatures, and (b) its proportional representation among flowering species across seasons and the corresponding seasonal temperatures. We tested whether, across pollination syndromes, (a) predicts (b).

## 2 Methods

### 2.1 Pollination Syndromes

Floristical data were retrieved from SynBioSys abundance releves from 2017 (Hennekens and Schaminee 2002). The species list was curated by updating taxonomic names to the World Checklist of Vascular Plants (Govaerts et al. 2021), using the “U.TaxonStand” R package (Zhang and Qian 2023). Exotic and invasive species (“neophytes”) were removed. Species were classified as native or non‐native to the Netherlands using Plant of the World Online (POWO 2021) and Euro + Med PlantBase (Euro+Med 2006). Subspecies, species aggregates, hybrid species, and taxa not identified to the species level were excluded from the analysis.

For each plant species, pollination syndrome was retrieved from BiolFlor (Kühn et al. 2004) determined based on available floral ecology literature and floral morphology. Specifically, this determination uses traits such as the accessibility of nectar combined with the depth of flowers compared to proboscis lengths of pollinator groups and direct observations of pollinators. Species that belong to multiple main pollination syndromes in Bioflor were ranked for simultaneously the multiple pollinator categories for the analysis. Because BiolFlor includes a large number of specific pollinator categories, we merged them into eight broader pollination syndromes based on taxonomic similarity (e.g., moths and butterfly are grouped under the category “Lepidoptera” and short‐tongued bees, medium‐tongued bees, and long‐tongued bees are all grouped under the category “Bees”) (see Table S1). While the utility of pollination‐syndrome classifications has been debated, some studies argue that floral traits do not always predict actual pollinators, Rosas‐Guerrero et al. (2014) show that syndromes reliably capture the main pollinator group for many species, particularly pollinator‐dependent taxa. Given the absence of comprehensive and standardized direct pollinator observations for most species, we consider this syndrome‐based grouping a pragmatic and justified approach for large‐scale comparative analyses, while acknowledging its limitations. We stress that these patterns of age structure do not reconstruct pollination mode through time, which would be impossible to for a local flora representing less than 1% of the global pool of extant species.

### 2.2 Phylogeny

A Dutch phylogenetic tree was made by using DNA sequences available on GenBank (Clark et al. 2016). This phylogeny represents an almost complete sample of species with DNA information available (Leclère et al. 2026). It is therefore a considerable improvement to the frequently used in ecological studies of large floras global phylogeny (Smith and Brown 2018), in which more than 20% of species from the region of our study were missing. Moreover, we also found numerous instances (34) of obviously wrong placement of species from our regional sample in the global phylogeny (Smith and Brown 2018), which makes it less reliable. For each taxon in our study, we included sequences of the nuclear ribosomal internal transcribed spacer (i.e., ITS) as well as at least one plastid marker (e.g., matK, rbcL, trnL‐trnF, or additional chloroplast loci when available). All sequences were aligned using MUSCLE option in MEGA 11 (Tamura et al. 2021) and later used in MrBayes v3.2.7 (Ronquist et al. 2012) to construct the tree. Input alignments were checked for gaps and ambiguous characters before tree inference. To improve resolution, the tree was reconstructed in two steps: phylogenetic inferences were first conducted for 36 subsets of taxonomically related species, defined at the order, family or genus level depending on clade size to balance subset size against the proportion of missing data, and individual subtrees were subsequently merged into the global phylogeny using the R packages, geiger (Pennell et al. 2014), ape (Paradis et al. 2004) and phytools (Revell 2024). We partitioned a concatenated matrix of all different genes combined for each of the 36 subsets of species and assigned GTR + Invariate + Gamma (a General Time Reversible model with a proportion of invariable sites and a gamma‐shaped distribution of rates across sites) substitution model per each partition. In each analysis, we used four chains in two runs. The number of generations in all analyses varied between one and five million, depending on the complexity of different analyses, and the burn‐in settings were set as by default (0.25). In all analyses, the average standard deviation of split frequencies decreased to values between 0.005 and 0.05 and we considered this level adequate for the purpose of our phylogenetic reconstructions. Posterior probabilities for specific nodes were verified for consistency with replicate runs. We used relaxed clock model and calibration of the stem nodes of all 36 subtrees based on the mean age estimates for these nodes from the tree produced by Hermant et al. (2012). Details of the dating analyses for the sub‐trees are specified in Leclère et al. (2026). The exact calibration priors for each subtree are fully documented in the 36 MrBayes input files provided in the dataset iDiv3600 (https://doi.org/10.25829/idiv.3600‐myr3t3), which maintains all basic data and details of the phylogenetic and dating analyses. The trees were summarized as majority‐rule consensus trees with PP 0.5 as the threshold. The nodes with PP < 0.5 were treated as unresolved. This tree is nearly fully resolved (i.e., ≈93%) and time‐calibrated, providing estimates of species divergence times, which was essential for assigning lineages to different time intervals considered in our study. The remaining 5 species without DNA information were included into the tree as polytomies at the crown nodes of corresponding genera. Remaining polytomies were treated as soft polytomies in the analysis, given the high resolution, their impact on stELD estimates is expected to be minimal. The tree includes all 1178 species representing the native Dutch angiosperm flora and considered in our analysis.

We generated a lineage‐through‐time (LTT) plot to visualize the temporal accumulation of angiosperm lineages in the regional flora. LTT curves were calculated from the dated phylogeny using the function ltt.plot() in the ape package (Paradis et al. 2004) in R Studio (R Core Team 2022). Geological time was plotted in millions of years before present, and node ages were extracted directly from the dated tree.

### 2.3 Phylogenetic Age Structure of Pollination Syndromes, Paleotemperatures

To quantify the proportional increase in numbers of lineages during a given epoch for a given pollination syndrome, we used standardized epoch‐specific lineage diversities (stELD). The stELD was calculated using the “ltteld” function implemented in Phylocom (Webb et al. 2008), which computes Epoch Lineages Diversity (ELD) by counting, for each 10 myr epoch and each pollination syndrome, the proportional increase in number of extant lineages whose stem ages fall within that interval (log2end–log2beginning). Phylocom then standardizes the ELD value of a given pollination syndrome within a given epoch by the null expectation across all pollination syndromes to produce stELD, a dimensionless index describing whether for a given pollination syndrome the proportional increase in number of lineages in a given epoch is higher (i.e., positive stELD value) or lower (i.e., negative stELD value) relative to the entire regional flora for the same epoch. stELD was chosen because it controls for differences in species richness across epochs and can be calculated across floras composed of hundreds of lineages, each only represented by a few species. This approach allows us to detect whether a particular pollination syndrome is over‐proportionately composed of lineages originating in specific epochs, providing a direct link between macroevolutionary history and ecological traits. We calculated stELD values for all 10‐million‐year intervals from 160 Ma to the present for each of our eight pollination syndromes. 10 Myrs was a compromise between the confidence that we have in individual branching events (preventing shorter intervals) and the temporal scale of variation of thermal environment (preventing longer individuals).

Global temperatures for different geological epochs were extracted from the literature (Scotese et al. 2021; Westerhold et al. 2020; Zachos et al. 2008; Jenkyns 2010), using the estimated Global Average Temperature (GAT) for each 10‐million‐year time interval.

To visualize how diversification patterns differ among pollination syndromes, we pruned the dated regional phylogeny to generate sub‐phylogenies for individual pollination syndromes using the ape package in R (Paradis and Schliep 2019). This also allowed inspection of whether syndrome‐specific thermal responses detected in the stELD analyses were driven by one species‐rich clade or whether they were distributed across multiple independent lineages.

### 2.4 Seasonal Representation of Pollination Syndromes, Seasonal Temperatures

We quantified the proportional representation of pollination syndromes across the flowering season in half‐month intervals. For each species, the seasonal median of flowering activity was calculated as the start of flowering +0.5 × flowering duration from BiolFlor (Kühn et al. 2004). For each pollination syndrome and half‐month interval, we calculated the number of species whose median flowering fell within that interval. Unsurprisingly, these numbers increased toward summer in all syndromes. We hence divided for each syndrome the number of species per half‐month interval by the total number of species to obtain a seasonal representation.

Mean temperatures of each half month were obtained from the KMNI Climate Normals 1991–2020 dataset (Royal Netherlands Meteorological Institute, accessed 2025).

### 2.5 Statistical Analysis

To assess whether stELD showed a systematic relationship with geological time, we used the stELD values for each 10‐Myr interval from 160 Ma to the present. Geological age (Ma) was treated as a continuous predictor, and a quadratic model was fitted to evaluate linear and nonlinear temporal trends. We verified error distributions using QQ plots and predicted vs. residual plots, and retained a Gaussian error distribution. Cook's distance and hat values did not identify any significant outliers, and rerunning the model without the one moderate outlier did not change levels of significance.

We examined how stELD varied with paleotemperature and pollination syndrome using lm models in RStudio (R Core Team 2022). Paleotemperature was treated as a continuous variable and modeled using both its linear term (temperature) and quadratic term (temperature^2^) (given predictions for increased stELD under both the highest and lowest paleotemperature). Pollination syndrome was treated as a categorical fixed factor with either three levels (insect, wind, water) or eight levels (bee, bumblebee, fly, wasp, Lepidoptera, beetle, wind, water), depending on the analysis.

For each pollination model, we fitted a Gaussian linear model of the form:

$$\text{stELD}=\beta_{0}+\beta_{1}T+\beta_{2}T^{2}+\beta_{3}P+\beta_{4}\left(T\times P\right)+\beta_{5}\left(T^{2}\times P\right)+\varepsilon ,$$

where T is paleotemperature, $T^{2}$ its quadratic term, and P the pollination syndrome, represented by separate parameters for each syndrome and for their interactions with temperature. All categorical predictors were coded with sum‐to‐zero contrasts (contr.sum), ensuring that Type III tests properly evaluated main effects after accounting for interactions (Maxwell and Delaney 2004). Model significance was assessed using Type III ANOVA implemented in the *car* package (Fox and Weisberg 2019), providing *F*‐statistics and *p*‐values for each term. We interpreted the main effects even in the presence of significant interaction terms as main effects were more significant than the interaction terms. Specifically, we extracted estimated marginal means (EMMs) and model predictions for each pollination syndrome across a range of paleotemperatures (14°C–24°C, at 1°C increments), using the *emmeans* package (Lenth 2025). These estimates were used to visualize temperature‐specific responses and to compare predicted stELD values among pollination syndromes across the paleotemperature gradient. Confidence intervals were obtained from model‐based predictions while holding other variables constant. We emphasize overall interaction patterns and temperature‐dependent trajectories and interpreted them based on t values (with wind as standard) and graphs (pairwise post hoc contrasts are little helpful, given their limited interpretability in the context of complex interaction models).

For the three‐category model, the same regression structure was used, but pollination was collapsed into “insect”, “wind”, and “water”. This allowed comparison between a coarse biotic–abiotic framework as it is inevitably used in the literature on global pattern (Stephens et al. 2023) and the more detailed eight‐category model. Model fit was evaluated using *R* ^*2*^ and adjusted *R* ^*2*^, albeit we stress that the two models cannot be directly compared given the very different error dfs (38 vs. 100).

Finally, to evaluate whether the paleotemperature‐dependent age structure of pollination syndromes aligns with contemporary thermal phenology and to provide a bridge between macroevolutionary and ecological timescales, we explored whether, across pollination syndromes, the relationship between paleotemperatures and stELD of pollination syndromes resembles the relationship between seasonal temperatures and seasonal representation of pollination syndromes. (1) We calculated a multiple regression for each of the 8 pollination syndromes explaining its stELDs of 16 epochs (each 10‐million‐years) by the corresponding paleotemperature and paleotemperature^2^. We verified error distributions using QQ plots and predicted vs. residual plots, and retained a Gaussian error distribution. We did not include age as such because the above analyses across all pollination types had shown that age is not maintained when paleotemperature is included in the model. We retained for each pollination syndrome the parameter estimates for paleotemperature and for paleotemperature^2^. (2) We calculated a multiple regression for each of the 8 pollination syndromes explaining its proportional representation during 15 half months (March to October) by the corresponding regional temperature and regional temperature^2^. Given that the dependent variable is a proportion, we used a beta logit model and a logit link function (Ferrari and Cribari‐Neto 2004). Since standard beta regression requires responses in (0, 1), zeros were replaced by a small positive constant (0.0001) to enable model fitting, a commonly used pragmatic approach when zeros represent detection limits rather than total, definite absence such as the absence of any single individual of given pollination type ever flowering in a given month (Ferrari and Cribari‐Neto 2004; Cribari‐Neto and Zeileis 2010; Douma and Weedon 2019; Warton and Hui 2011). We also include time (= number of half months from 3 to 10) as such because models across all pollination syndromes had shown that time is maintained when seasonal temperature is included in the model. Including time among others corrects for possible biases such as the fact that flowering under still cold, late‐winter temperatures is only possible for perennial plants, many of which are wind‐pollinated (such as grasses, many trees). We again retained for each pollination syndrome the parameter estimates for seasonal temperature and for seasonal temperature^2^. (3) We finally correlated, across pollination syndromes, (i) the parameter estimates for paleotemperatures and for seasonal temperature, (ii) the parameter estimates for paleotemperatures^2^ and for seasonal temperature^2^.

## 3 Results

**FIGURE 2:**
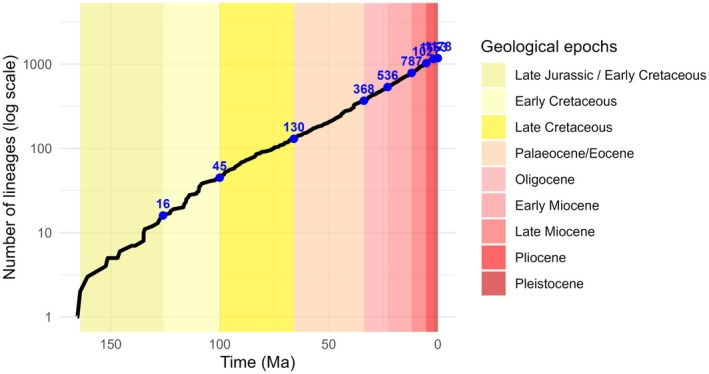
Lineages‐through‐time (LTT) plot for the regional angiosperm phylogeny. The blue dots indicate the number of lineages at the transition between two geological epochs.

We constructed a lineages‐through‐time (LTT) plot of the dated phylogeny that shows a gradual accumulation of angiosperm lineages from the Mesozoic to the present, with an increase in the rate of lineage accumulation beginning in the Late Cretaceous and continuing throughout the Cenozoic, without any major shift in the rate of accumulation (Figure 2).

**FIGURE 3:**
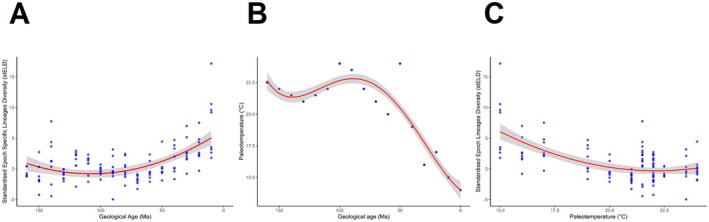
(A) Temporal pattern of standardized epoch‐specific lineages diversity (stELD). Points represent observed stELD values for individual pollination syndromes across 10‐Myr time intervals. The red curves show the predicted stELD from a quadratic model of stELD as a function of geological age (age and age^2^ terms) with the shaded gray area indicating the 95% confidence interval. The time axis has been reversed for better clarity. (B) Reconstruction of paleotemperature over geological time. The blue dots represent the paleotemperature estimate retrieved from literature (Scotese et al. 2021; Westerhold et al. 2020; Zachos et al. 2008; Jenkyns 2010) for each 10‐Myr time intervals. The red curve shows the polynomial trend with the shaded gray area indicating the 95% confidence interval. The time axis has been reversed for better clarity. (C) Model‐based temporal pattern of standardized epoch‐specific lineages diversity (stELD). Points represent observed stELD values for individual pollination syndromes across 10‐Myr time intervals. The red curve shows the predicted stELD trajectory from a quadratic model of stELD as a function of paleotemperature (temperature and temperature^2^ terms) with the shaded gray area indicating the 95% confidence interval.

We found that stELD shows a nonlinear relationship with age (Figure 3A; see Figure S1 for patterns by pollination syndrome), which combined with the paleotemperature‐age relationship from the literature (Figure 3B) results in an even more distinct relationship between paleotemperature and stELDs (Figure 3C), with the statistical model indicating higher stELD values toward both the coldest and warmest intervals, and reduced values at intermediate temperatures. A model including age, age^2^, temperature, and temperature^2^ showed that only temperature (linear and quadratic) significantly explained stELD, while age had no effect (Table S2).

When pollination syndromes were grouped into insect‐, wind‐, and water‐pollination syndromes categories, a Type III ANOVA revealed a significant overall effect of pollination syndrome on stELD (Table 1 left). stELD showed a negative relationship with paleotemperature and a positive relationship with paleotemperature^2^, indicating a U‐shaped association with temperature. However, significant interactions between pollination syndrome and paleotemperature, as well as between pollination syndrome and paleotemperature^2^, indicate that the direction and strength of temperature effects differed among pollination categories. In particular, species with wind‐pollination syndrome showed a strong decrease in stELD with increasing temperature and a pronounced U‐shaped response (Figure 5A), whereas species with insect‐pollination syndrome displayed practically no temperature dependence (Figure 5A). Water‐pollination syndrome was too rare to be interpretable (*n* = 8), and we refrain from further detailing (or discussing) results on water‐pollination syndrome.

**TABLE 1:** Statistical effects of pollination syndrome, paleotemperature, the quadratic effect of paleotemperature, and their interactions on lineage diversification (stELD) with all insect pollination grouped (left; Adjusted *R* ^2^ = 0.67) and all insect pollination split (right; Adjusted *R* ^2^ = 0.38). Shown are *F*‐statistics and associated *p*‐values for each term. Note that the adjusted *R* ^2^ is not directly comparable between models given different dfs.

Effect	SS	df	MS	*F*	*p*	SS	df	MS	*F*	*p*
	3‐category model					8‐category model				
Intercept	109.510	1	109.510	24.12	< 0.001	125.42	1	125.42	28.81	< 0.001
Pollination syndrome	73.58	2	36.79	8.10	0.001	94.5	7	13.50	3.10	0.005
Temperature	86.08	1	86.08	18.96	< 0.001	100.50	1	100.50	23.08	< 0.001
Temperature^2^	69.12	1	69.12	15.22	< 0.001	81.50	1	81.50	18.72	< 0.001
Pollinator × Temperature	61.23	2	30.615	6.74	0.003	87.95	7	12.56	2.89	0.009
Pollinator × Temperature^2^	51.97	2	25.98	5.72	0.006	81.47	7	11.64	2.67	0.014
Error	172.55	38	4.54			435.35	100	4.35		

**FIGURE 4:**
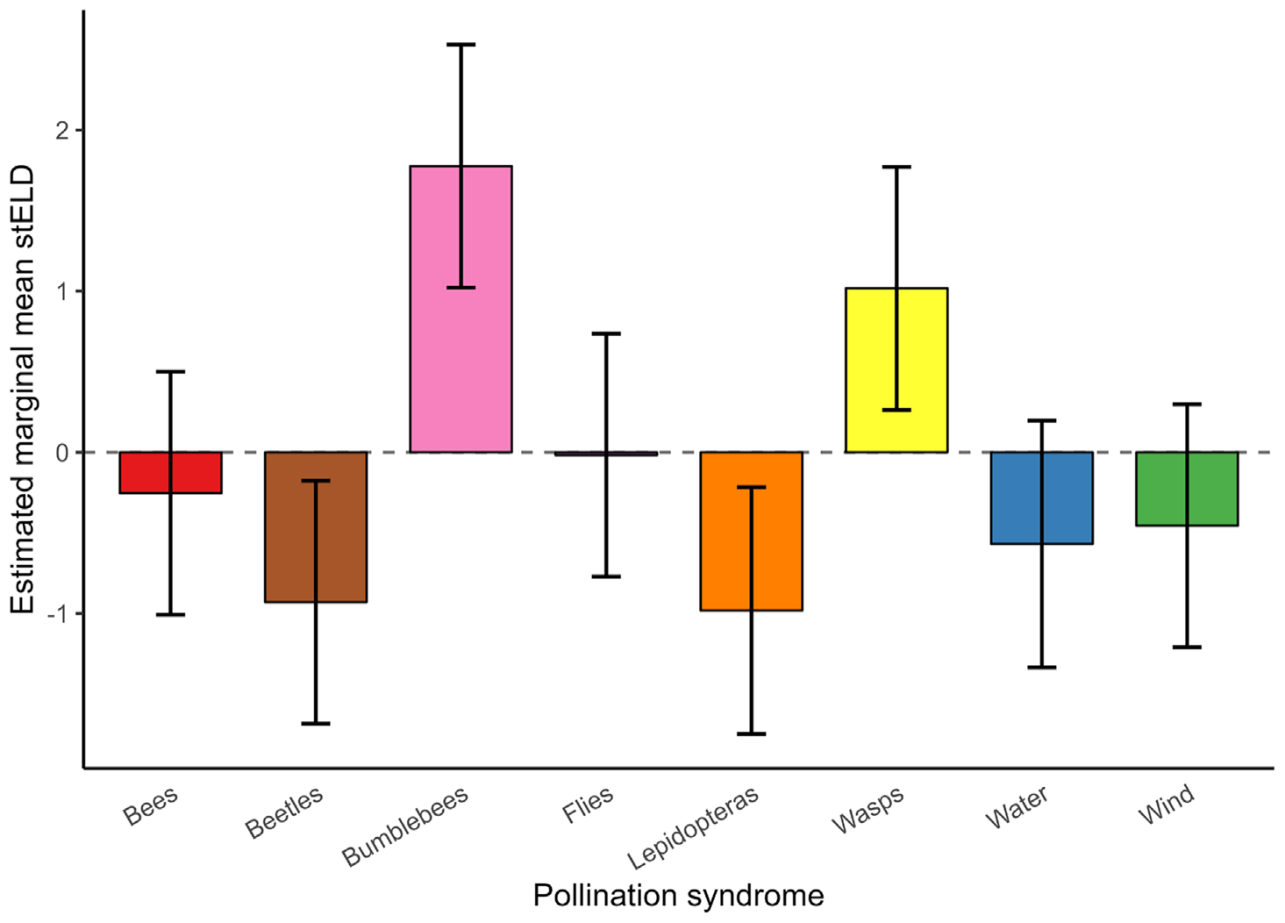
Main effect of pollination syndrome on stELD. Mean stELD values (±95% confidence intervals) are shown for the eight pollination syndromes across all paleotemperatures.

**FIGURE 5:**
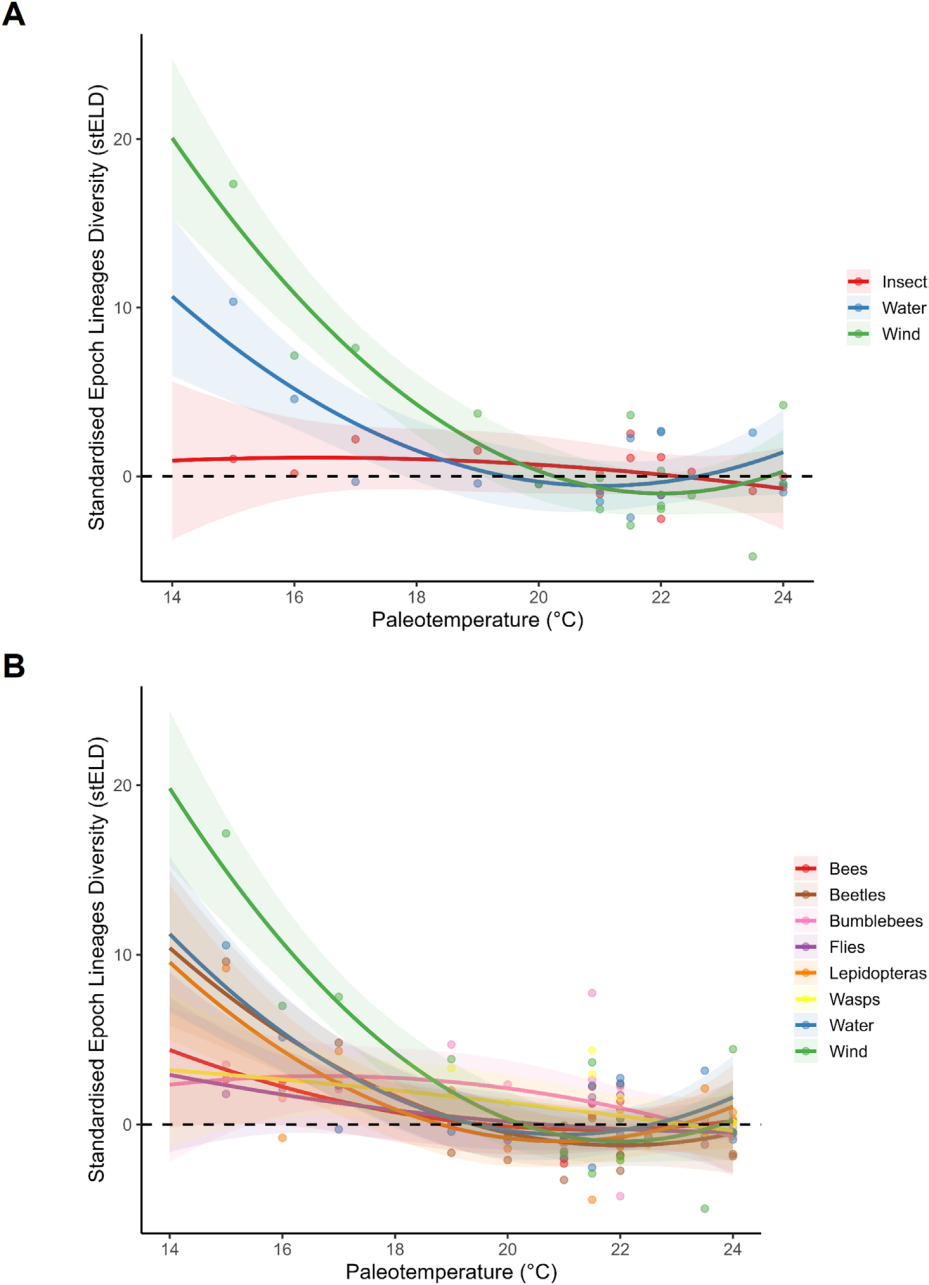
Relationship between standardized epoch‐specific lineages diversities (stELD) and paleotemperature across pollination syndromes for (A) the 3‐category model and (B) the 8‐category model. Colored lines show the predicted stELD for each pollination syndrome based on a model including linear and quadratic effects of temperature and their interactions with the pollination syndrome. The shaded areas represent the 95% confidence intervals. Points represent observed stELD values, and colors correspond to pollination syndrome identity. See Table 1 for (A) and (B) statistical analyses, left and right respectively.

When considering the eight pollination syndromes, our model using pollination syndrome, temperature, temperature^2^, and the interaction between pollination syndrome × temperature and pollination syndrome × temperature^2^ (Table 1 right) showed that all the predictors are significant. The main effect of pollination syndrome revealed pronounced differences in mean stELD among syndromes (Figure 4). Species with bumblebee‐pollination syndrome showed the highest estimated marginal mean stELD, followed by species with wasp‐pollination syndrome. Species with beetle‐ and lepidoptera‐pollination syndrome exhibited lowest mean stELD values. Inspection of individual interaction terms (t‐values, relative to wind pollination as the reference category) further indicated that species with bumblebee‐ and wasp‐pollination syndrome differed significantly from species with wind‐pollination syndrome in both the direction and curvature of their responses to both paleotemperature and paleotemperature^2^ (*p* ≤ 0.059 and *p* < 0.05, respectively): species with bumblebee and wasp pollination syndromes showed a significantly more positive stELD trend with temperature (compared to the wind pollination) and a significantly more negative one with temperature^2^, resulting in comparatively somewhat higher stELD values at the intermediate temperatures dependence overall (Figure 5B, global mean paleotemperatures of approximately 14–17/−23/> 23°C). In contrast, during coldest epochs, species with wind‐pollination syndrome showed higher stELD values than other pollination syndromes (Figure 5B). As paleotemperatures increased, contrasts among pollination syndromes progressively diminished (Figure 5B; Figure S2).

**FIGURE 6:**
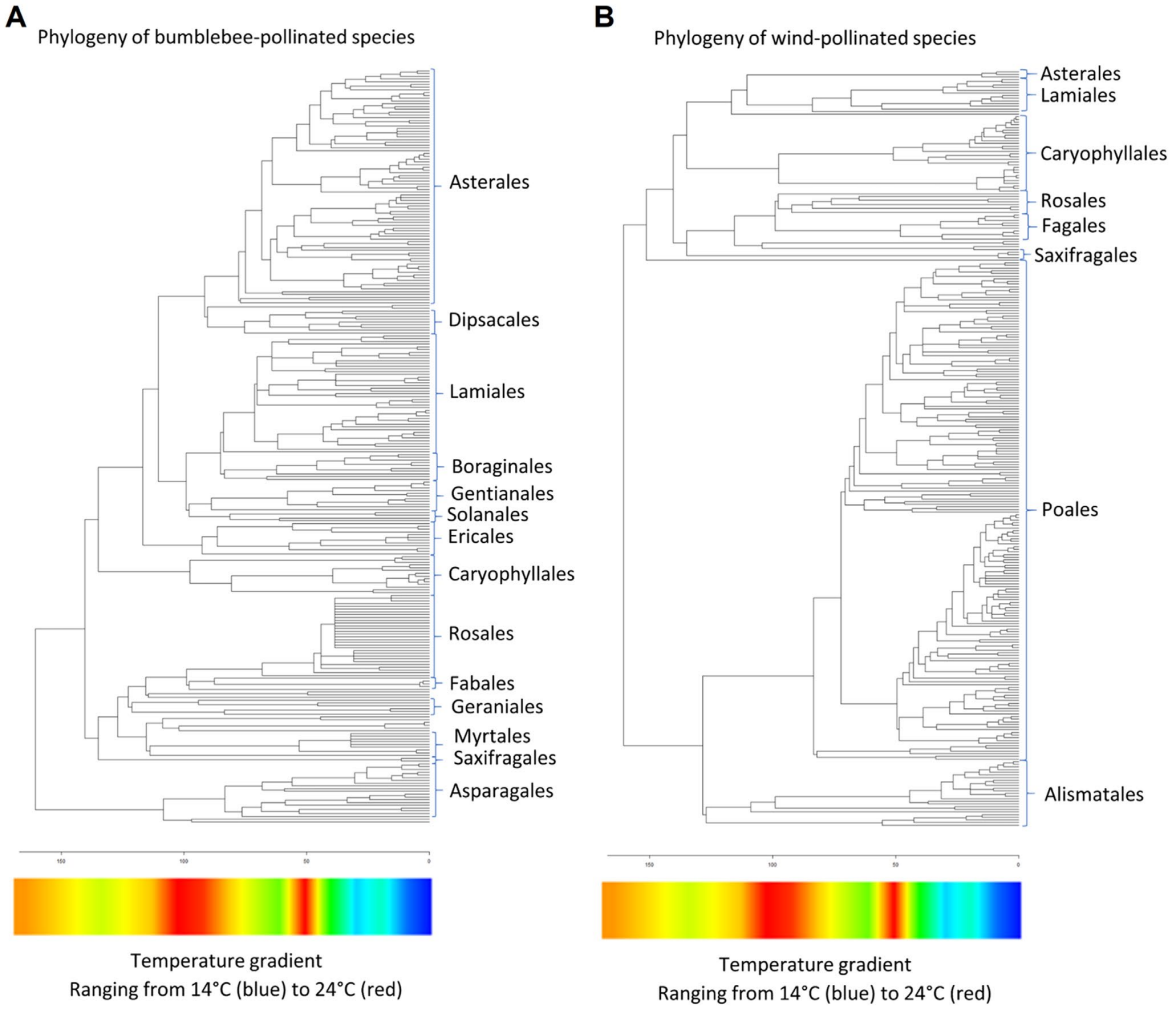
Phylogenetic trees of (A) species of bumblebee‐pollination syndrome lineages and (B) species of wind‐pollination syndrome. Major angiosperm orders are written next to the trees. A time scale and a temperature gradient are displayed below the trees.

To visualize the phylogenetic patterns underlying the analysis of stELDs in Table 1 and Figure 5B, we plotted phylogenetic trees for species of different pollination syndromes. We here (Figure 6) present the trees for syndromes that have the most contrasting patterns of stELD, wind‐pollination syndrome and bumblebee‐pollination syndrome (for remaining ones, please see Figure S3–S8). The age structure of species of bumblebee‐pollinated syndrome showed branching across a wide range of paleotemperature, including both warm and intermediate (Figure 6A). By contrast, the age structure of species of wind‐pollinated syndrome showed some limited branching under warm paleoclimates and substantial recent branching during coldest intervals (Figure 6B). While this pattern is inevitably strongly driven by the very numerous and entirely wind‐pollinated Poales, it appears not restricted to them (comparatively major radiation in Caryophyllales, comparatively rapid radiation of Lamiales). Overall, the patterns indicate that temperature‐dependent stELD signatures observed in the regression analyses arise from multiple independent lineages within each syndrome rather than an individual diversification event of a single lineage.

**FIGURE 7:**
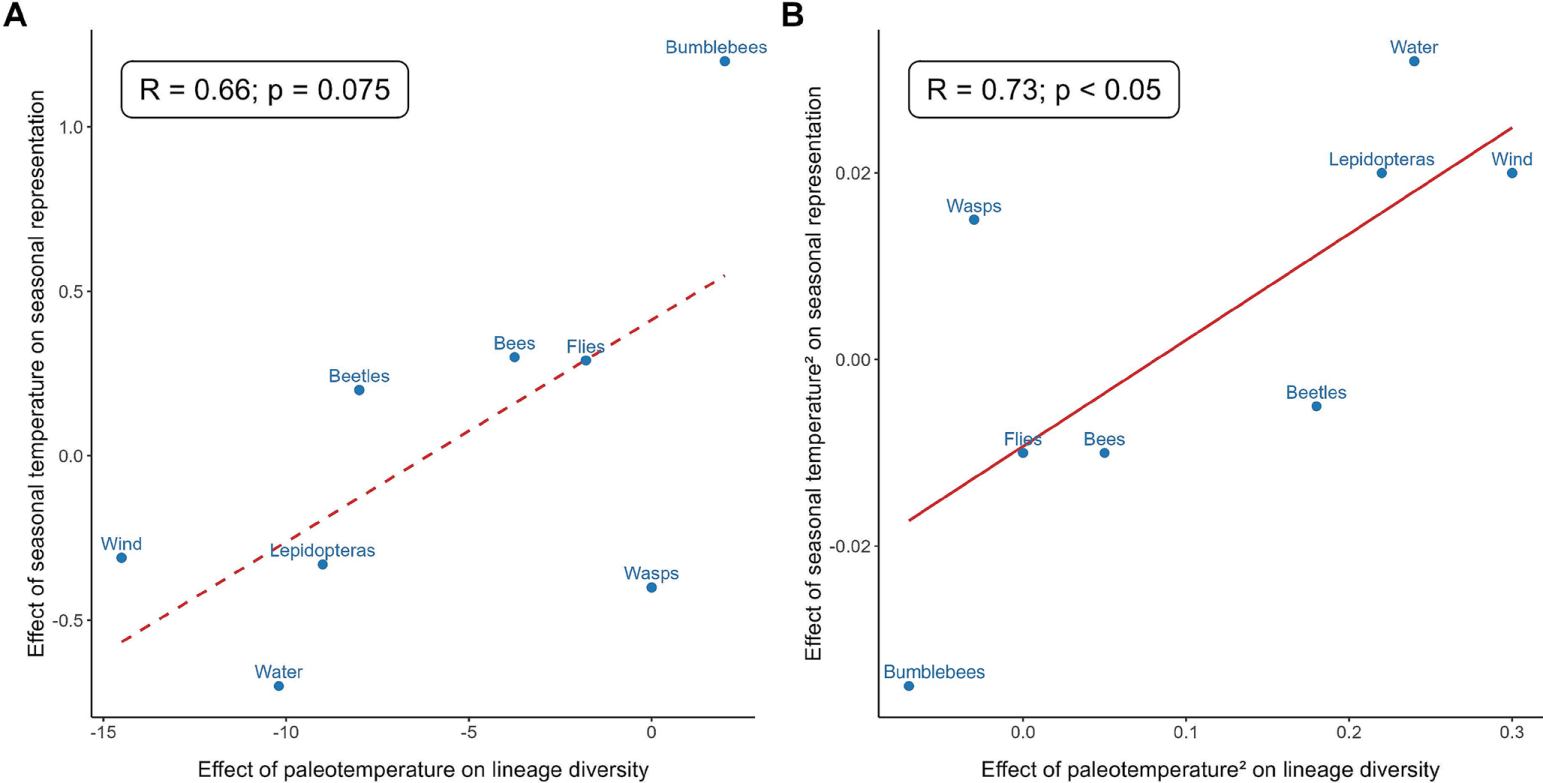
Relationship between the statistical effect of seasonal temperature on seasonal representations of pollination syndromes (Y) and the statistical effect of paleotemperature on standardized epoch‐specific lineage‐diversities (stELD, X) of the same pollination syndromes: Effect of temperature (A) and temperature^2^ (B). A continuous regression line indicates significance, and a dotted line indicates marginal significance.

Across pollination syndromes, coefficients describing the quadratic response of stELD to paleotemperature were positively correlated with the quadratic response of seasonal representation to present‐day temperature (*R* = 0.73, *p* < 0.05; despite only 8 categories, Figure 7B). Linear coefficients showed a similar but weaker trend (*R* = 0.66, *p* = 0.07; Figure 7A). Thus, syndromes whose age structure reflected warmest paleotemperatures tended to exhibit warmest seasonal flowering temperature niches, and in tendency the same is true for relationships to coldest temperatures.

## 4 Discussion

Our analyses show that the age structure of the regional angiosperm flora is strongly associated with paleotemperature and pollination strategy. stELDs revealed a pronounced nonlinear relationship with paleotemperature, with the highest stELD values occurring during both the coldest and warmest geological epochs, and low stELD values during intermediate temperatures. Pollination syndromes significantly modulated these patterns: species with wind‐pollination syndromes showed the highest stELD value during the coldest intervals, whereas species with insect‐pollination syndrome displayed heterogeneous responses that only became apparent when insect pollination was resolved into distinct syndromes. These relationships of phylogenetic age structure of pollination syndromes to paleotemperature resembled those of seasonal representation to seasonal temperature.

We portrayed the age structure through a lineage‐through‐time (LTT) plot as well as through stELDs. The LTT plot indicates that lineages dating to ancient, intermediate, and more recent geological intervals are all similarly represented in the present‐day angiosperm flora of the region. However, LTT plots are inherently constrained to show a monotonic increase toward the present (McCarthy et al. 2021), and their interpretation has long been debated (McCarthy et al. 2021; Morlon 2014; Pennell et al. 2012). Approximately linear trends are often viewed as biologically uninformative or difficult to relate to underlying diversification processes. In this context, the LTT in our study primarily serves as a descriptive backdrop rather than a direct test of hypotheses. By contrast, the stELD approach quantifies the relative representation of lineages dating to individual geological epochs in the modern flora, independent of adjacent epochs and standardized against a null expectation (Bartish et al. 2016, 2020). This allows deviations from uniform lineage accumulation to be identified and directly related to paleotemperature and pollination strategy. The subsequent regression analyses across epochs then revealed overarching temporal patterns that could not be recovered from classical LTT plots alone, notably a relatively low representation of lineages dating to intermediate epochs and the highest representation of lineages dating to the most recent epochs. This U‐shaped age structure within a small region is largely consistent with global reconstructions of angiosperm diversification (Zuntini et al. 2024; Condamine et al. 2020; Sauquet and Magallón 2018), which show an even more pronounced drop during intermediate epochs (Zuntini et al. 2024; Condamine et al. 2020; Sauquet and Magallón 2018). This consistency suggests that the present age structure within a small region may be influenced by macroevolutionary diversification of lineages across the planet, rather than being entirely the result of biased colonization of that region by certain lineages and within‐region extinction (Donoghue 2008; Ramírez‐Barahona et al. 2020).

The recent half of the U‐shaped relationship between stELD and time might result from the lower impact of extinctions on recent branching patterns. However, we found that this U‐shaped relationship resulted from an even stronger relationship between stELD and paleotemperatures (which replace for time when included as explanatory variables into the same model). This strong U‐shaped relationship between paleotemperature and stELD indicates that geological intervals characterized by either warmest or, in particular, coldest climates are overrepresented among lineages persisting in the present‐day regional flora, whereas intervals with intermediate temperatures are underrepresented. Lineages dating back to warm intervals likely reflect the legacy of global angiosperm radiations that occurred under high temperatures and elevated CO_2_, conditions known to promote plant growth, ecological opportunity, and lineage origination (Sauquet and Magallón 2018; Zuntini et al. 2024; Zachos et al. 2008; Scotese et al. 2021). The persistence of these lineages in the regional flora suggests long‐term survival rather than continuous regional diversification (Donoghue 2008; Crisp et al. 2009). This is consistent with global reconstruction showing that many angiosperm clades that originated during the mid‐Cretaceous warming persisted through climatic transitions without major radiations post‐Cretaceous (Magallón et al. 2015; Zuntini et al. 2024; Tank et al. 2015). By contrast, the high stELD associated with cold intervals likely reflects the more recent origin and accumulation of lineages adapted to cooler climates, which have experienced fewer subsequent climatic transitions and thus a higher probability of persistence to the present (Condamine et al. 2020; Strömberg 2011; Zuntini et al. 2024). In contrast, lineages originating during intermediate climatic regimes do not coincide with the major Angiosperm radiations but may already have been exposed to repeated environmental shifts between warm and cold conditions, increasing extinction or turnover, and reducing their representation in the modern flora (Sauquet and Magallón 2018; Magallón et al. 2015; Zuntini et al. 2024). We stress that this remains an interpretation: stELD reflects the age structure of lineages currently persisting in the regional flora, which may result from elevated speciation rates during particular paleoclimatic intervals, differential survival of lineages that originated then, or a combination of both processes. The history of speciation and extinction cannot be inferred from the age structure of a regional flora harboring 1% of the global richness, and only part of the global temperature gradient. This limitation applies to all paleotemperature‐stELD associations reported in this study and not only to the warm‐interval pattern discussed here. However, the consistency between our regional stELD pattern and global diversification reconstructions suggests that stELD captures, at least in part, the imprint of diversification under paleoenvironments rather than solely the result of recent ecological convergence within ancient lineages into a particular climate.

We found that significantly structured stELDs variation through their interaction with paleotemperature. Finding differences in present age structures of different pollination syndromes and statistically explaining them by paleotemperatures is no proof of a true causal relationship; in particular, it lacks fossil evidence for a pollination syndrome really dating back to these ages. We will hence below compare our observations with what is known from the fossil record. We will indeed show convergence with the literature studying the fossil record or reconstructing histories of entire lineages. Nevertheless, pollination syndromes might have disappeared in lineages in which they once originated and appeared in other lineages long after their origin, rendering correlations between age structures of present pollination syndromes and paleotemperatures difficult to interpret. The interpretation of high stELD from an old epoch in fact implicitly assumes some degree of conservatism in the distribution of pollination modes across lineages, and indeed such signal has been found ((Xiang et al. 2023); Prinzing et al. 2021 for flowering traits phenology). High stELDs from more recent epochs, in contrast, are consistent with recent convergence of pollination syndromes among lineages, for which there is also evidence (Schiestl and Johnson 2013).

When pollination syndromes were grouped coarsely into insect‐, wind‐, and water‐pollination syndrome categories we found the highest stELD values at the coldest paleotemperatures, consistent with temperature‐independent pollen transfer and reduced insect activity under cold conditions (Culley et al. 2002; Friedman and Barrett 2008; Rafferty and Ives 2011). This result is also consistent with global observations on inflorescence types (Wang et al. 2025) and seed dispersal modes (Jin et al. 2026), which suggest that evolution of pollination mutualism between plants and animals is suppressed in coldest climates globally. The fossil record also suggests diversification under coldest conditions (Linder et al. 2018). By contrast, grouping all species with insect‐pollination syndrome into a single category, obscured thermal patterns in stELD, suggesting that opposing responses among insect pollination syndromes cancel out at this coarse level. Trees of species with water‐pollination syndrome, overall, showed very low stELDs, likely reflecting their small size and ecological specialization within the flora (Armbruster 2014; Rosas‐Guerrero et al. 2014). Together, the three‐category model suggests a broad biotic–abiotic turnover driven by paleotemperature, with abiotic pollination favored under coldest intervals with little change in insect pollination. This is consistent with global‐level studies (e.g., Stephens et al. 2023), which however, do not differentiate between different insect‐pollinator system for lack of available data.

Disaggregating pollination syndromes into eight categories revealed strong contrasts that were hidden in the three‐category model. Species with a wind‐pollinated syndrome again dominated among lineages dating to the coldest vs. intermediate paleotemperatures, consistent with the recurrent expansion of open habitats and the diversification of anemophilous clades during late Cenozoic cooling (Strömberg 2011). By contrast, insect‐pollinated species partitioned into pollinator groups with distinct baseline stELD values and differed in association between stELD and paleotemperature. This pattern suggests that pollinator identity might have shaped diversification trajectories through geological time. Together, the eight‐category model demonstrates that insect pollination encompasses a diversity of thermal niches and evolutionary histories that are obscured when collapsed into a single “insect” category. Methodologically, resolving pollination syndromes at finer scales is only possible in floras for which traits like types and accessibility of pollinator reward and flower depth are known for all species. Moreover, identifying the consequences of different syndromes of insect pollinators requires regional floras with highly resolved, species‐level phylogenies, which allow lineage age structure to be traced into recent, climatically cold epochs where effects are strongest. Dissecting phylogenetic trends in a regional flora across geological time therefore provides a powerful framework for detecting macroevolutionary signals within megadiverse clades that would remain inaccessible using global phylogenies or coarse ecological categories.

Species with lepidoptera‐pollination syndrome showed the highest stELD values at the warmest paleotemperatures, consistent with the strong dependence of lepidoptera activity on ambient temperature and solar radiation. As ectothermic insects, butterflies require warm and sunny conditions for sustained flight and nectar foraging, as their pollination efficiency declines under cool conditions (Liao et al. 2020; Mesler and Mabry 2024). In addition, characteristic lepidoptera‐pollination traits, such as visually conspicuous flowers and tubular or nectar‐concealing corollas, are most effective under warm conditions that promote lepidoptera visitation and mobility (Fenster et al. 2004; Willmer 2011). This ecological signal is consistent with fossil‐based reconstructions showing a steep increase in Lepidoptera family diversity around 50 million years ago, coinciding with the Eocene thermal maximum (Peris and Condamine 2024). By contrast, phylogenetic trees of species with bumblebee‐ and wasp‐pollination syndromes branched more than other trees at intermediate paleotemperatures; they branched slightly less than other trees at high paleotemperatures and distinctly less at coldest paleotemperatures. Low stELDs at coldest temperatures is clearly contrary to our expectation in particular for bumblebees. Despite bumblebees being able to forage at relatively low ambient temperatures, by employing facultative endothermy and social thermoregulation (Heinrich 1974; Heinrich 2013), the observed pattern suggests that low stELD values at the coldest paleotemperatures may reflect limited recent diversification of bumblebee‐pollinated species during the youngest geological intervals, rather than a lack of thermal suitability (Hines 2008; Condamine and Hines 2015; Santos Júnior et al. 2022). The absence of a dominant temperature signal in bumblebee‐pollinated species is similarly consistent with fossil evidence showing a gradual diversification of Hymenoptera across the Paleogene without a single dominant climatic episode (Peris and Condamine 2024), suggesting that bees diversified under a broad range of climatic conditions rather than tracking a particular thermal optimum. We also note that some flowers may be pollinated by bumblebees without having a bumblebee syndrome: this syndrome reflects exclusion of non‐bumblebees more than facilitation of bumblebees. Exclusion might be less needed during coldest temperatures.

Species with lepidoptera‐pollination syndrome also showed elevated stELD values associated with the coldest paleotemperature, just like those of fly and beetle‐pollination syndromes. For lepidopteras and flies, these cold‐biased signatures likely reflect the ecology of the plants they pollinate rather than extreme physiological cold tolerance in the pollinators themselves. Many lepidoptera‐ and fly‐pollinated angiosperms flower in cool, open, or seasonal temperate habitats where short daily warming windows permit intermittent pollination (Shreeve 1986; Dennis 1993; Arroyo et al. 1982), and diptera and lepidoptera increasingly dominate pollination networks toward higher elevations and latitudes (Totland 1994; Arroyo et al. 2013). Several temperate lepidoptera lineages also diversified during late Cenozoic cooling and the expansion of temperate biomes (Condamine and Hines 2015; Rainford and Mayhew 2015), potentially reinforcing cold‐associated stELD signals. Beetle pollination also exhibited high stELDs at cold paleotemperatures, consistent with the ability of many beetles to forage by walking rather than sustained flight and to remain active at lower temperatures (Gottsberger 1990; Bernhardt 2000; Willmer 2011). This pattern is further corroborated by fossil evidence showing a marked increase in Coleoptera family diversity during Neogene cooling (Peris and Condamine 2024). In addition, beetle pollination dominates several early‐diverging angiosperm clades (Thien et al. 2009) that persisted through Neogene cooling (Sauquet and Magallón 2018), combining physiological cold tolerance with long‐term lineage survival.

Together, the observed age structures of different pollination syndromes are consistent with a hypothesis of a thermal hierarchy in the diversification of pollination strategies, with butterflies associated with both the warmest and coldest epochs, bumblebees and wasps at intermediate temperatures, bees, flies, and beetles at colder temperatures, and the coldest intervals favoring abiotic wind pollination where flowering conditions restrict biotic pollination entirely. We stress that this hypothesis cannot be interpreted globally based on average, less than 1% of the global richness of the lineages involved, and that present regional age structures may depend on processes other than past origin, such as convergence of pollination syndrome in the meantime. But for the single pollination syndrome (among those listed) for which present data permit such global tests, wind pollination, these tests confirm our above hypothesis (Stephens et al. 2023). We further acknowledge that the Netherlands represents a single temperate flora with a distinct post‐glacial history, and that the relative contribution of recent biogeographic filtering versus deeper macroevolutionary signal to the patterns we describe may differ in floras with other glacial or biogeographic histories. Whether the age‐structure‐pollination relationships we identify generalize to other temperate or tropical floras remains to be tested.

The positive association between paleotemperature‐dependent stELD coefficients and seasonal‐temperate dependent representation of flowering syndromes suggests that pollination syndromes occupy relatively conserved thermal niches. Pollination syndromes with an age structure suggesting diversification during warmest paleoclimates also dominate flowering activity during the warmest parts of the modern season, and syndromes with age structures peaking under coldest paleotemperatures tend to dominate under coolest conditions. This cross‐scale consistency aligns with temperature dependence of pollinator flight performance, metabolic heat production, and foraging phenology (Heinrich 1974; Willmer and Stone 2004; Settele et al. 2016) as well as with niche conservatism in flowering phenology (Davies et al. 2013; Wolkovich et al. 2014; Prinzing et al. 2021). Thus, pollination mode appears to impose thermal and phenological constraints that operate from ecological to geological timescales, linking macroevolutionary filtering of lineages to contemporary community assembly.

Together, our results show that the age structure of regional angiosperm flora retains detectable signatures of paleoclimate and pollination strategy. By linking age structure to pollination syndrome and seasonal temperature, we show that rather than reflecting a uniform accumulation of lineages through time, different pollination modes show distinct temporal distributions of surviving branches, indicating that present‐day floras are assembled from lineages that became established under different climatic conditions. The strong representation of lineages associated with the coldest geologic intervals further suggests that recent increases in branch numbers may partly reflect the timing of diversification of plant groups linked to pollination syndromes that expanded under cooler climates, rather than temperature acting solely as a present‐day ecological filter. In this sense, regional floras appear to preserve a record of climatic history and the evolutionary timing of pollination strategies jointly shaped which lineages persist today in regional biomes.

## Author Contributions

**Thomas Leclère:** conceptualization (lead), data curation (equal), formal analysis (equal), methodology (equal), visualization (lead), writing – original draft (lead), writing – review and editing (equal). **Igor Bartish:** conceptualization (equal), data curation (equal), formal analysis (equal), methodology (equal), supervision (equal), writing – original draft (supporting), writing – review and editing (equal). **Pille Gerhold:** conceptualization (supporting), supervision (equal), writing – original draft (supporting), writing – review and editing (equal). **Andreas Prinzing:** conceptualization (lead), data curation (equal), formal analysis (equal), methodology (equal), supervision (lead), writing – original draft (supporting), writing – review and editing (lead).

## Funding

This work was supported by Eesti Maaülikool (P210165PKKK).

## Conflicts of Interest

The authors declare no conflicts of interest.

## Supporting information


**File S1:** ece374150‐sup‐0001‐SupplementaryfileS1.xlsx.

**File S2:** ece374150‐sup‐0002‐SupplementaryfileS2.csv.

**File S3:** ece374150‐sup‐0003‐SupplementaryfileS3.txt.

**Table S1:** Main pollinator categories in BiolFlor and how they were merged in the present study. Each color represents one pollinator system.
**Table S2:** Output of the linear model explaining stELD based on age and temperature and their quadratic terms.
**Figure S1:** Temporal patterns of standardized epoch lineages diversity (stELD) separated by pollination syndrome. Points represent stELD values for each 10‐Myr interval. Red curves show fitted temporal trends for each pollination system. Some pollination systems exhibit distinct temporal diversification trajectories, including early or late diversification peaks, while others follow the broader temporal pattern without pronounced deviations. Axes are scaled independently to highlight within‐group variation.
**Figure S2:** Relationship between standardized epoch lineages diversity (stELD) and paleotemperature for each pollination syndrome. Points represent stELD values at 10‐Myr intervals, and colored lines show the predicted diversification response based on a model including linear and quadratic temperature terms and their interactions with the pollination system. Panels are scaled independently.
**Figure S3:** Phylogenetic tree of bee‐pollinated species in our study with a time scale and a temperature gradient.
**Figure S4:** Phylogenetic tree of beetle‐pollinated species in our study with a time scale and a temperature gradient.
**Figure S5:** Phylogenetic tree of butterfly‐pollinated species in our study with a time scale and a temperature gradient.
**Figure S6:** Phylogenetic tree of fly‐pollinated species in our study with a time scale and a temperature gradient.
**Figure S7:** Phylogenetic tree of wasp‐pollinated species in our study with a time scale and a temperature gradient.
**Figure S8:** Phylogenetic tree of water‐pollinated species in our study with a time scale and a temperature gradient.

## Data Availability

Floral trait data were obtained from the BiolFlor database (https://www.ufz.de/index.php?en=38567), and community composition data were obtained from the SynBioSys database (https://www.synbiosys.alterra.nl/synbiosysnl/). DNA sequences used for phylogenetic analyses were obtained from GenBank (accession numbers provided in File S2). Derived datasets summarizing species by trait are provided in File S3. The phylogenetic tree generated in this study, along with the DNA sequence alignments, MrBayes input files, and calibration priors for all 36 sub‐trees, are deposited in the iDiv data repository (iDiv3600, https://doi.org/10.25829/idiv.3600‐myr3t3).
