# Supplementary material for: Signals of Paleotemperature in the Age Structure and Seasonal Distribution of Pollination Syndromes Across a Temperate Region
Source: Ecol Evol. 2026 Aug 11;16(8):e74150. doi: 10.1002/ece3.74150 (PMC13460016; doi:10.1002/ece3.74150)
Supplement: Supplementary file 1 [file ECE3-16-e74150-s004.docx]

**Supplementary files**

**Table S1.** Main pollinator categories in BiolFlor and how they were merged in the present study. Each color represents one pollinator system.

| **Main pollinator category in BiolFlor** | **Main pollinator category in the present study** |
| --- | --- |
| Bees | Bees |
| Short-tongued bees |  |
| Long-tongued bees |  |
| Medium-tongued bees |  |
| Hymenopteres |  |
| Butterflies | Lepidoptera |
| Moths |  |
| Syrphids | Flies |
| Muscidae |  |
| Bombylides |  |
| Flies |  |
| Very small dipteres |  |
| Beetles | Beetles |
| Icheunmonidae | Wasps |
| Small ichneumonide |  |
| Wasps |  |
| Bumblebees | Bumblebees |
| Water | Water |
| Wind | Wind |

**Table S2.** Output of the linear model explaining stELD based on age and temperature and their quadratic terms.

| Coefficients | Estimate | Std.Error | t value | p value |
| --- | --- | --- | --- | --- |
| **intercept** | **5.971e+01** | **1.697e+01** | **3.520** | **<0.05** |
| **temperature** | **-5.394e+00** | **1.740e+00** | **-3.100** | **<0.05** |
| **temperature²** | **1.222e-01** | **4.132e-02** | **2.958** | **<0.05** |
| age | -1.546e-02 | 4.130e-02 | -0.374 | 0.709 |
| age² | 9.339e-05 | 1.999e-04 | 0.467 | 0.641 |

**Figure S1.** Temporal patterns of standardised epoch lineages diversity (stELD) separated by pollination syndrome. Points represent stELD values for each 10-Myr interval. Red curves show fitted temporal trends for each pollination system. Some pollination systems exhibit distinct temporal diversification trajectories, including early or late diversification peaks, while others follow the broader temporal pattern without pronounced deviations. Axes are scaled independently to highlight within-group variation.

**Figure S2.** Relationship between standardised epoch lineages diversity (stELD) and paleotemperature for each pollination syndrome. Points represent stELD values at 10-Myr intervals, and coloured lines show the predicted diversification response based on a model including linear and quadratic temperature terms and their interactions with the pollination system. Panels are scaled independently.

**Figure S3.** Phylogenetic tree of bee-pollinated species in our study with a time scale and a temperature gradient.

**Figure S4.** Phylogenetic tree of beetle-pollinated species in our study with a time scale and a temperature gradient.

**Figure S5.** Phylogenetic tree of butterfly-pollinated species in our study with a time scale and a temperature gradient.

**Figure S6.** Phylogenetic tree of fly-pollinated species in our study with a time scale and a temperature gradient.

**Figure S7.** Phylogenetic tree of wasp-pollinated species in our study with a time scale and a temperature gradient.

**Figure S8.** Phylogenetic tree of water-pollinated species in our study with a time scale and a temperature gradient.
